# Perilipin 2 Stabilizes Lipid Droplets and Coordinates Mitochondrial Fatty Acid Flux and ER Stress Adaptation in *Apostichopus japonicus*

**DOI:** 10.3390/ijms27114859

**Published:** 2026-05-28

**Authors:** Hong Fan, Jintao Yu, Wenhao Wang, Zhimeng Lv, Si Zhu, Chenghua Li

**Affiliations:** 1 State Key Laboratory of Agricultural Products Safety, Ningbo University, Ningbo 315211, China; 15669178030@163.com (H.F.); lvzhimeng@nbu.edu.cn (Z.L.); 2 School of Business, Ningbo University, Ningbo 315211, China; yjt6636@126.com; 3 Yantai Marine Economic Research Institute, Yantai 264000, China; wangwenhao1128@163.com; 4 Laboratory for Marine Fisheries Science and Food Production Processes, Qingdao National Laboratory for Marine Science and Technology, Qingdao 266237, China

**Keywords:** lipid droplet, perilipin 2, mitochondria, endoplasmic reticulum, *Apostichopus japonicus*

## Abstract

Perilipins (PLINs) are lipid droplet-associated proteins that regulate lipid storage, mobilization, and metabolism, yet their roles in invertebrates remain poorly characterized. This study aimed to investigate the evolutionary conservation and functional adaptation of PLIN2 in the sea cucumber *Apostichopus japonicus*. Phylogenetic analysis placed *A. japonicus* PLIN2 within the PLIN2 clade, forming an echinoderm-specific branch distinct from vertebrate PLIN2s. Structural prediction revealed an N-terminal PAT domain containing an amphipathic helix that was required for lipid droplet targeting, as deletion of this region abolished its localization to lipid droplet. Functionally, PLIN2 abundance positively correlated with lipid droplet formation, and its knockdown reduced triacylglycerol accumulation while upregulating lipolysis-related genes. Pull-down and co-immunoprecipitation assays identified interactions between PLIN2 and the endoplasmic reticulum protein ERP44, as well as the mitochondrial protein TRXR2, suggesting a role in lipid droplet–organelle coupling. Consistently, disruption of the PLIN2-TRXR2 module impaired fatty acid transfer from lipid droplets to mitochondria, leading to suppressed β-oxidation and decreased ATP production. In addition, PLIN2 mediates the protective role of lipid droplets against *Vibrio splendidus*-induced ER stress. Together, these findings establish *A. japonicus* PLIN2 as a multifunctional lipid droplet-associated protein that coordinates lipid droplet stability with organelle communication, energy metabolism, and ER homeostasis.

## 1. Introduction

Lipids are fundamental determinants of growth performance, reproductive capacity, and stress resilience in aquaculture species. Abnormal lipid storage and mobilization are frequently associated with reduced growth efficiency, physiological dysfunction, and increased vulnerability to environmental stressors and pathogens [1,2,3]. A deep understanding of the mechanisms governing lipid storage and mobilization is therefore important for improving aquaculture productivity and animal health. Within cell, lipid droplets serve as the primary sites of lipid storage, consisting of a neutral lipid core mainly composed of triacylglycerols (TAG), and cholesteryl esters, with smaller amounts of diacylglycerols and monoacylglycerols, surrounded by a phospholipid monolayer embedded with associated proteins [4,5]. Although once viewed as inert lipid depots, lipid droplets are now recognized as dynamic organelles that actively participate in cellular lipid metabolism, metabolic modulation, stress responses, and innate immunity [6,7]. These diverse functions are closely tied to the ability of lipid droplets to interact both physically and functionally with other organelles. Accumulating evidence shows that lipid droplets establish contacts sites with the endoplasmic reticulum (ER), mitochondria, lysosomes, peroxisomes, and even the nucleus [8,9].

The functions of lipid droplets are largely governed by their associated proteins, which play critical roles in lipid droplet biogenesis, stability, and lipid mobilization. Among these, the perilipin (PLIN) family represent a key group of lipid droplet coat proteins. PLINs are members of the PAT protein family, defined by a conserved N-terminal PAT domain, 11-amino acid (11-mer) repeats, and a carboxy-terminal domain [10]. In mammals, five perilipin members (PLIN1-PLIN5) have been identified, each exhibiting distinct tissue distributions and regulatory functions. PLIN2 is the only isoform that is constitutively and ubiquitously expressed and has been widely implicated in lipid droplet formation and stabilization [11,12]. Overexpression of PLIN2 increases cellular triglyceride level and promotes lipid droplets formation in various cell types, whereas loss of PLIN2 accelerates lipolysis [13,14]. Mechanistically, PLIN2 restricts access of lipases, such as adipose triglyceride lipase (ATGL), hormone-sensitive lipase (HSL), and monoacylglycerol lipase (MGL) to the droplet surface, thereby inhibiting excessive lipolysis and facilitating the deposition of lipid droplets [15]. Despite the growing understanding of perilipin function in vertebrates, their evolutionary conservation and physiological roles in invertebrates remain poorly characterized. Comparative genomic and evolutionary analyses suggest that the expansion of the PLIN family occurred primarily in vertebrates, whereas invertebrates generally possess fewer PLIN homologs [10]. This limited diversification implies that invertebrate PLINs may integrate multiple functions that are partitioned among different isoforms in vertebrates.

Echinoderms represent an important lineage of deuterostome invertebrates and provide valuable insights into the evolution of lipid metabolic regulation. The sea cucumber *Apostichopus japonicus* is an ecologically and economically important marine species in China and Southeast Asia. Lipid metabolism plays essential roles in its growth, reproduction, and environmental adaptation [16,17]. However, the molecular mechanisms underlying lipid droplet dynamics in this species remain largely unclear. Genome-wide analysis revealed that only PLIN2 was identified in *A. japonicus*. Therefore, this study aimed to investigate the molecular characteristics, evolutionary features, and potential functional roles of PLIN2 in lipid droplet regulation in *A. japonicus*. Specifically, we examined its involvement in lipid droplet formation, stability, and mobilization, as well as its potential role in mediating organelle interactions under physiological and stress conditions. These findings are expected to provide new insights into the evolution and functional diversification of PLIN proteins in invertebrates.

## 2. Results

### 2.1. Multiple Sequence Alignment of PLINs

**Figure 1:**
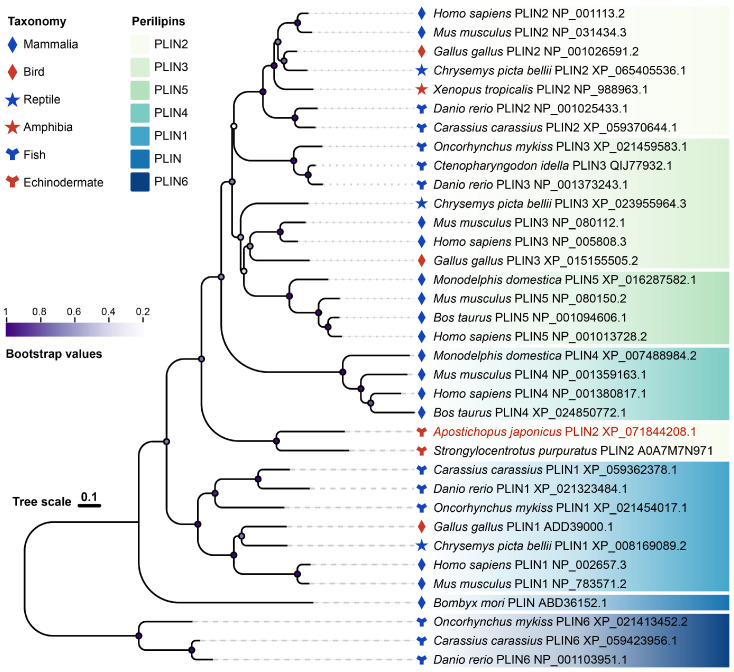
Phylogenetic analysis of perilipin (PLIN) family proteins across representative species. A maximum-likelihood phylogenetic tree was constructed using amino acid sequences of PLIN family members from vertebrates and invertebrates. Different PLIN family members (PLIN1-PLIN6) are indicated by distinct color blocks on the right. Major taxonomic groups are denoted by symbols, including mammals, birds, reptiles, amphibians, fish, and echinoderms. *Apostichopus japonicus* PLIN2 is highlighted in red. Node support is represented by a color gradient corresponding to bootstrap values. The scale bar represents the number of amino acid substitutions per site.

In the phylogenetic tree, *A. japonicus* PLIN2 clustered within the PLIN2 clade and was positioned close to *Strongylocentrotus purpuratus* PLIN2, indicating that the two echinoderm sequences are sister groups and share a recent common ancestor. This echinoderm-specific branch was clearly separated from vertebrate PLIN2 members, including those from mammals (*Homo sapiens*, *Mus musculus*), birds (*Gallus gallus*), reptiles (*Chrysemys picta*), amphibians (*Xenopus tropicalis*), and fishes (*Danio rerio*, *Carassius carassius*) (Figure 1). The topology therefore suggests that PLIN2 has been evolutionarily conserved across metazoans, while the echinoderm lineage has diverged early from the vertebrate PLIN2 lineages.

### 2.2. PLIN2 Binds Lipid Droplets via the PAT Domain

**Figure 2:**
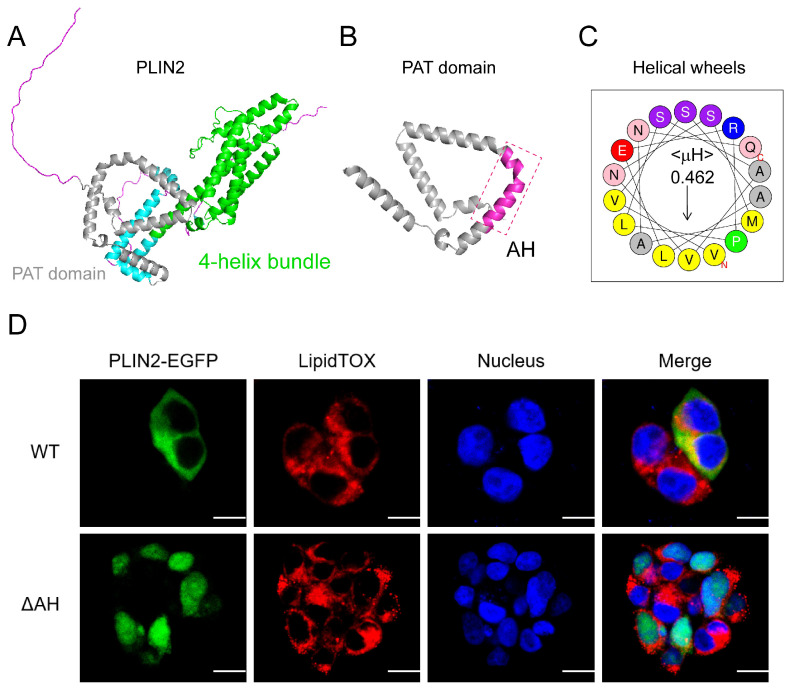
PLIN2 binds lipid droplets via the PAT domain. (**A**) Predicted three-dimensional structure of PLIN2. The N-terminal PAT domain is highlighted in gray, and the C-terminal region forms a four-helix bundle (shown in green). (**B**) Enlarged view of the PAT domain, with the predicted amphipathic helix (AH) highlighted in magenta (dashed box). (**C**) Helical wheel projection of the predicted AH of PLIN2 generated using the HeliQuest tool. The hydrophobic moment (μH) represents the amphipathic nature of the helix. (**D**) Subcellular localization of PLIN2 and the AH-deficient variant (ΔAH). HEK293T cells were transfected with PLIN2-EGFP (WT) or ΔAH-EGFP (ΔAH) (green) for 24 h, followed by fixation and staining with LipidTOX (red) to label lipid droplets and DAPI (blue) for nucleus. Images were acquired by confocal microscopy. Scale bar = 10 μM. PLIN2, perilipin 2.

Although *A. japonicus* PLIN2 shares low overall sequence similarity with vertebrate PLIN2, both are predicted to adopt a similar structure, featuring an N-terminal PAT domain composed of three α-helices arranged in a triangular formation (shown in gray) and a C-terminal four-helix bundle (shown in green) (Figure 2A). We next investigated whether PLIN2 contains amphipathic helix (AH) sequence features that mediate its localization to lipid droplets. Potential α-helix regions in PLIN2 protein were identified using the Phyre2 protein structure prediction server, and each α-helix was subsequently analyzed with HeliQuest to determine its helical wheel orientation. The α-helix structure was selected primarily when hydrophobic and polar residues segregated into two distinct faces, with hydrophobic moments (μH) > 0.4, hydrophobicity (H) between 0.3 and 0.6, and a net charge slightly positive. Based on these criteria, PLIN2 was predicted to contain a potential AH region within the PAT domain with a μH of 0.462, H of 0.375, and a net charge of 0 (Figure 2B,C). To determine whether this AH is required for lipid droplet binding, an AH-deficient PLIN2 variant (ΔAH) was constructed. Loss of AH altered its subcellular localization, with the ΔAH mutant no longer targeting lipid droplets but instead being exclusively localized to the nucleus (Figure 2D).

### 2.3. PLIN2 Stabilizes Lipid Droplets by Restraining Lipolytic Activation

**Figure 3:**
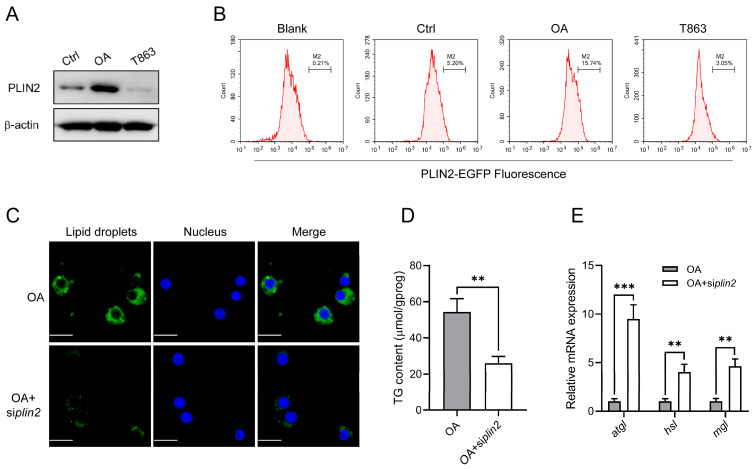
PLIN2 stabilizes lipid droplets by restraining lipolytic activation. (**A**) Coelomocytes were treated with OA or T863 for 12 h. Protein expression of PLIN2 was determined by Western blot, with β-actin as the loading control. Data represent three independent experiments. (**B**) HEK293T cells were transfected with the PLIN2-EGFP expression plasmid. After 24 h, cells were treated with OA or T863 for 12 h, harvested, and analyzed by flow cytometry using the FITC channel to quantify EGFP fluorescence intensity. (**C**–**E**) Coelomocytes were transfected with siNC or si*plin2*. After 24 h, cells were incubated with 500 μM OA for 12 h, followed by fixation and staining with BODIPY 493/503 (green) to label lipid droplets and DAPI (blue) for nucleus. Images were acquired by confocal microscopy. Scale bar = 10 μM (**C**). Cellular triglyceride content was measured (**D**), and relative mRNA expression of *atgl*, *hsl*, and *mgl* was detected by qRT-PCR (**E**). Data from at least three independent biological replicates (mean ± SD). Significantly different experimental groups: ** *p* < 0.01, *** *p* < 0.001 by Student’s *t* test. *atgl*, adipose triglyceride lipase; *hsl*, hormone-sensitive lipase; *mgl*, monoacylglycerol lipase; PLIN2, perilipin 2; OA, oleic acid.

The protein level of PLIN2 in coelomocytes was significantly increased following OA treatment but decreased in response to T863 treatment, an inhibitor of triacylglycerol biosynthesis (Figure 3A). Consistently, fluorescence of PLIN2-EGFP in HEK293T cells was enhanced after OA treatment but dismissed upon exposure to T863 (Figure 3B). To assess the correlation between PLIN2 and lipid droplets abundance, we introduced siRNA targeting PLIN2. Knockdown of PLIN2 led to a marked reduction in lipid droplet abundance (Figure 3C) and TAG content induced by OA treatment in coelomocytes (Figure 3D), implying that PLIN2 plays an important role in stabilizing lipid droplets. As expected, the expression of lipolysis-related genes, including *atgl*, *hsl*, and *mgl*, was significantly upregulated following PLIN2 knockdown (Figure 3E).

### 2.4. PLIN2 Interacts with the Endoplasmic Reticulum (ER) and Mitochondrial Proteins

**Figure 4:**
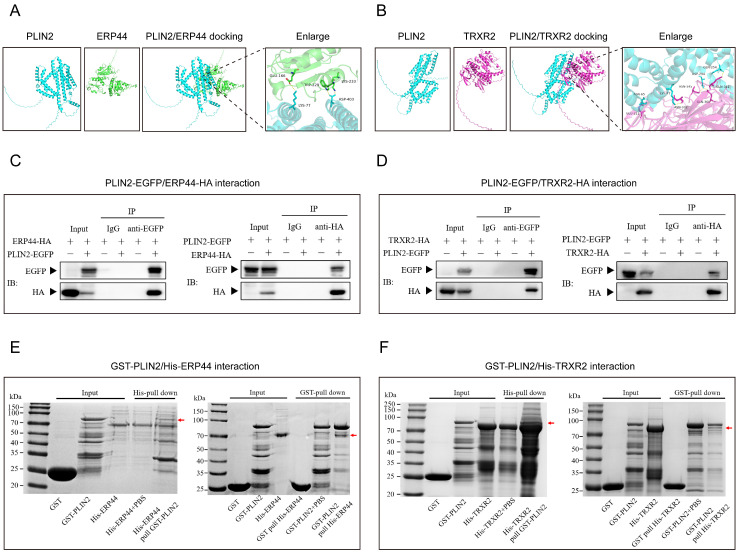
PLIN2 interacts with ERP44 and TRXR2. (**A**,**B**) Predicted interactions of PLIN2 with ERP44 and TRXR2 based on AlphaFold3 modeling, visualized using PyMOL2. (**C**,**D**) Co-immunoprecipitation validation in HEK293T cells. Cells cultured in 10 cm dishes were co-transfected with PLIN2-EGFP and ERP44-HA or TRXR2-HA plasmids for 48 h. IP was implemented with anti-EGFP or anti-HA beads. IB was conducted with anti-HA and anti-EGFP antibodies, respectively; mouse IgG was used as the control. Data represent three independent biological replicates. (**E**,**F**) In vitro pull-down validation. Recombinant GST-PLIN2, His-ERP44, and TRXR2 were expressed in *E. coli*. His pull-down was performed by incubating His-ERP44 or His-TRXR2 with GST-PLIN2, followed by capture with His-affinity resin. Reciprocal GST pull-down assays were performed using GST-Sepharose resin. Bound proteins were analyzed by SDS-PAGE. The red arrow indicates the protein band pulled down after the interaction. ERP44, endoplasmic reticulum resident protein 44; PLIN2, perilipin 2; TRXR2, thioredoxin reductase 2, mitochondrial.

PLINs have been reported to play a crucial role in tethering lipid droplets to various intracellular organelles, including ER and mitochondria, thereby contributing to the maintenance of cellular homeostasis. As the sole PLIN family member identified on lipid droplets in coelomocytes, PLIN2 may participate in mediating these organelle interactions. To test this possibility, a recombinant GST-tagged PLIN2 fusion protein was generated and used in a pull-down assay to identify potential interacting proteins from total proteins of coelomocytes. Mass spectrometry analysis identified several candidate interacting proteins, with the top five highest-scoring proteins listed in Supplemental Table S2. Notably, one ER-resident protein ERP44 and one mitochondrial protein TRXR2 were among the top-ranked candidates. Potential interactions between PLIN2 and ERP44 or TRXR2 were further analyzed using AlphaFold3 and visualized with PyMOL2. The results suggested that PLIN2-ERP44 and PLIN2-TRXR2 complexes are capable of forming binding pockets stabilized by hydrogen bonds (Figure 4A,B). Coimmunoprecipitation assay was also preformed to validate these interactions. Results showed that PLIN2 interacts with both ERP44 and TRXR2 (Figure 4C,D). In addition, reciprocal pull-down assays using His- and GST-tagged proteins further confirmed these interactions (Figure 4E,F).

### 2.5. PLIN2-TRXR2 Module Promotes Lipid Droplets-to-Mitochondria Fatty Acid Flux

**Figure 5:**
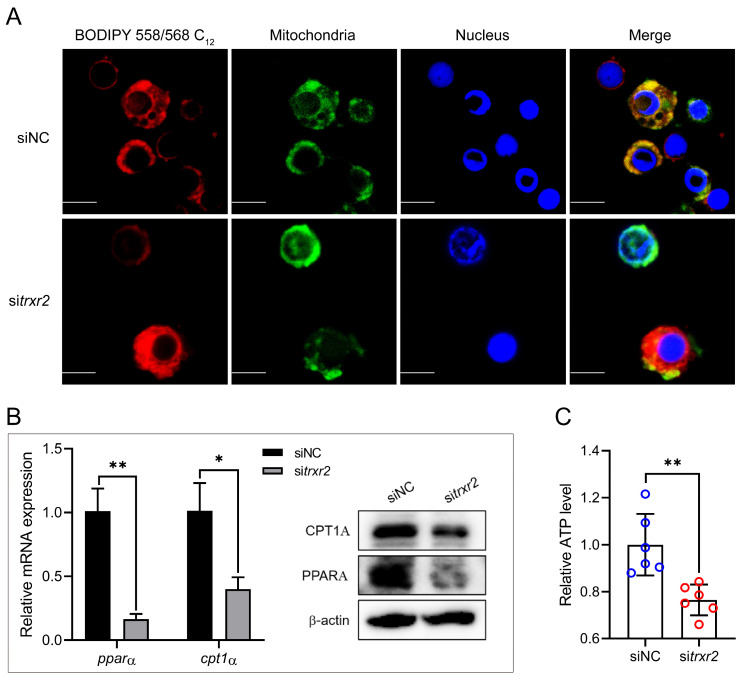
PLIN2-TRXR2 module promotes lipid droplets-to-mitochondria fatty acid flux. (**A**) Coelomocytes were transfected with siNC or si*trxr2* for 24 h, followed by treatment with 500 μM oleic acid (OA) for 12 h. Cells were then incubated with BODIPY 558/568 C_12_ (red) for 30 min (pulse) to label lipid droplet-associated fatty acids. After washing with PBS, cells were incubated in Leibovitz’s L-15 medium for 6 h (chase). Cells were then fixed and stained with Mito-Tracker Green and DAPI (blue). Images were acquired by confocal microscopy. Scale bar = 5 μM (**B**,**C**) Coelomocytes were transfected with siNC or si*trxr2* for 24 h, followed by treatment with 500 μM OA for 12 h. Cells were then incubated in Leibovitz’s L-15 medium for an additional 6 h. Relative mRNA and protein expression of PPARA and CPT1A (**B**) was detected by qRT-PCR and WB (**B**). Intracellular ATP content was determined according to the manufacturer’s protocol (**C**). Data from at least three independent biological replicates (mean ± SD) or representative data. Significantly different experimental groups: * *p* < 0.05, ** *p* < 0.01 by Student’s *t* test. CPT1A, O-palmitoyltransferase 1 (CPT1A); PPARA, peroxisome proliferator-activated receptor alpha; PLIN2, perilipin 2; TRXR2, thioredoxin reductase 2, mitochondrial.

Lipid droplet-mitochondria contacts facilitate the transfer of fatty acids to mitochondria. To determine whether the PLIN2-TRXR2 module is involved in this process, coelomocytes were subjected to TRXR2 knockdown concomitantly with OA treatment to induce lipid droplet formation and PLIN2 expression. A pulse-chase assay using the fluorescent fatty acid analog BODIPY 558/568 C_12_ (Red C_12_) was then performed to trace the transfer of fatty acids. At 0 h chase, Red C_12_ predominantly localized to lipid droplets (Supplementary Figure S2). After a 6 h of chase in nutrient-depleted medium, an increased signal was observed in mitochondria, indicating fatty acid transfer. Notably, knockdown of TRXR2 significantly reduced mitochondrial Red C_12_ accumulation (Figure 5A), suggesting impaired fatty acid flux to mitochondrial. Fatty acids trafficked from lipid droplets to mitochondria are metabolized via β-oxidation to generate ATP. Consistently, TRXR2 knockdown suppressed the expression of key genes involved in the fatty acid β-oxidation pathway, including carnitine O-palmitoyltransferase 1 (CPT1A) and peroxisome proliferator-activated receptor alpha (PPARA) (Figure 5B). As a consequence, inhibition of β-oxidation resulted in a significant reduction in ATP levels (Figure 5C).

### 2.6. PLIN2-Dependent Lipid Droplet Organization Mitigates Vibrio Splendidus-Induced ER Stress

**Figure 6:**
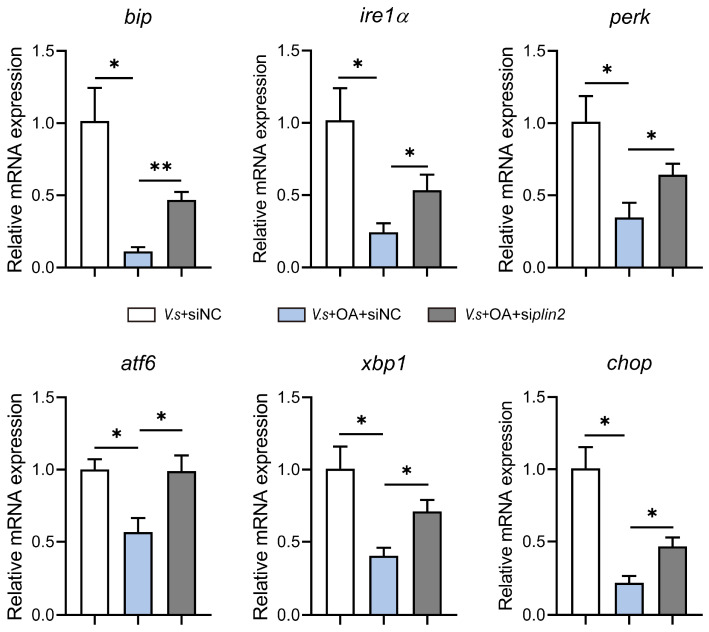
PLIN2-dependent lipid droplet organization mitigates *Vibrio splendidus*-induced ER stress. Coelomocytes were transfected with siNC or si*plin2* for 24 h, followed by infection with *V. splendidus* (MOI = 10) for 12 h in the absence or presence of 500 μM OA. Relative mRNA expression of *bip*, *ire1α*, *perk*, *atf6*, *xbp1*, and *chop* was detected by qRT-PCR. Data are presented as the mean ± SD from three independent biological replicates. Significantly different experimental groups: * *p* < 0.05, ** *p* < 0.01 by one-way ANOVA. *atf6*, cyclic AMP-dependent transcription factor atf-6 alpha; *bip*, endoplasmic reticulum chaperone bip; *chop*, DNA damage-inducible transcript 3 protein; *ire1α*, serine/threonine-protein kinase/endoribonuclease ire1α; OA, oleic acid; *perk*, eukaryotic translation initiation factor 2-alpha kinase 3; *plin2*, perilipin 2; *xbp1*, x-box-binding protein 1.

Our previous study demonstrated that *V. splendidus* infection induces ER stress in the coelomocytes of *A. japonicus*. Notably, a pronounced accumulation of lipid droplets was also observed in coelomocytes during infection [18,19]. Given the close association between lipid droplets and the ER, along with the essential role of PLIN2 in lipid droplet formation, we next examined its potential involvement in the ER stress response. The results showed that OA-induced lipid droplets formation significantly suppressed the mRNA expression of ER stress-related genes, including *bip*, *ire1α*, *perk*, *atf6*, *xbp1*, and *chop*. In contrast, knockdown of PLIN2 restored the expression of these genes, suggesting that PLIN2 mediates the protective effect of lipid droplets against *V. splendidus*-induced ER stress (Figure 6).

## 3. Discussion

Lipid droplets are highly conserved organelles that play central roles in cellular energy metabolism, organelle communication, and stress adaptation across eukaryotes [20,21,22]. Their functional diversity is largely governed by PLIN family, which exhibits lineage-specific expansion and specialization in different taxa [12,23]. In mammals, distinct PLINs regulate lipid storage, mobilization, and inter-organelle crosstalk; however, the evolutionary conservation and functional adaptation of PLIN proteins in invertebrates remain poorly understood.

In the current, only one perilipin gene (PLIN2) was presented in *A. japonicus*. Phylogenetic analysis of the PLIN family revealed clear clustering of subtypes into distinct clades, consistent with their evolutionary diversification. *A. japonicus* PLIN2 was positioned within the PLIN2 clade and grouped most closely with *Strongylocentrotus purpuratus* PLIN2, forming an echinoderm-specific branch that was clearly separated from vertebrate PLIN2 homologs in mammals, birds, reptiles, amphibians, and fishes. This topology indicates that the PLIN2 lineage originated prior to the divergence of deuterostomes and has been evolutionarily conserved, while echinoderm PLIN2 followed an independent trajectory reflecting unique lipid metabolic demands of marine invertebrates.

Although *A. japonicus* PLIN2 exhibits limited overall sequence identity with vertebrate PLIN2 proteins, structural predictions suggest a conserved fold, comprising an N-terminal PAT domain and a C-terminal four-helix bundle. Several 11-amino acid amphipathic helices (11-mer AHs) were detected within the *A. japonicus* PLIN2 sequence; however, these helices do not display the canonical 11-mer repeats typically associated with lipid droplet-binding motifs. Instead, an AH region within the PAT domain appears to play a dominant role in lipid droplets targeting, as deletion of this region abolished the association of *A. japonicus* PLIN2 with lipid droplets. This observation is consistent with previous studies showing that the loss of 11-mer repeats in PLIN3 does not prevent lipid membrane targeting, whereas its binding to artificial lipid droplets is mediated by interactions between the PAT domain and membrane diacylglycerol [24,25]. In the present study, HEK293T cells were used as a convenient and highly transfectable heterologous system for subcellular localization analyses. Nevertheless, species-specific differences in intracellular trafficking, lipid metabolism, and protein interaction dynamics between mammalian HEK293T cells and sea cucumber coelomocytes may influence the precise localization behavior of *A. japonicus* PLIN2. Therefore, further validation in primary coelomocytes or other physiologically relevant systems would help strengthen the biological relevance and generalizability of the present findings.

PLIN proteins coat lipid droplets and play central roles in regulating lipid metabolism. Recent studies have demonstrated that PLIN2 is required for lipid droplet stabilization [14], and our findings are consistent with this notion. Specifically, knockdown of *A. japonicus* PLIN2 abolished the OA-induced increase in lipid droplet abundance and TAG content in coelomocytes. Furthermore, neutral lipases appear to be involved in the effects of PLIN2 on cellular TAG homeostasis, as PLIN2 knockdown significantly upregulated the expression of lipolysis-related genes, including *atgl*, *hsl*, and *mgl*. Our observations complement previous studies showing that overexpression of PLIN2 in embryonic kidney cells limits lipase access to lipid droplets [15,26], whereas loss of PLIN2 promotes lipolysis in hepatocytes [27] and myotubes [28]. Taken together, our results indicate that *A. japonicus* PLIN2 functions to protect TAG stored within lipid droplets from neutral lipases-mediated lipolysis, thereby contributing to lipid droplet stability.

Increasing evidence indicates that lipid droplets are not inert lipid storage organelles but dynamic hubs that communicate extensively with other cellular compartments [7,9,29]. Such inter-organelle communication is largely mediated by proteins coating the lipid droplet surface, among which the PLIN family represents one of the most prominent and best-characterized regulators [10,12]. In the current study, our results provide evidence that PLIN2 interacts with proteins associated with both the endoplasmic reticulum and mitochondria, including ERP44 and TRXR2, suggesting that PLIN2, together with these organelle-associated proteins, may play an expanded role in coordinating communication between lipid droplet and other intracellular compartments. Indeed, in coelomocytes treated with OA to induce PLIN2 expression, knockdown of TRXR2 markedly impaired the accumulation of lipid droplet-derived fatty acids within mitochondria, indicating a disruption of lipid flux from lipid droplets to mitochondria. This disruption was accompanied by the downregulation of β-oxidation regulator, including CPT1A and PPARA, and a significant reduction in mitochondrial ATP production. These results highlight the functional importance of the PLIN2-TRXR2 module in facilitating lipid droplet-mitochondria coupling and maintaining cellular energy homeostasis. Due to spectral overlap between Red C_12_ and lipid droplet fluorescent probes, reliable simultaneous quantification of Red C_12_ distribution between these organelles could not be accurately achieved under our experimental conditions. Therefore, lipid droplet staining was not included in the pulse-chase imaging analysis shown in Figure 5 to avoid potential bias caused by fluorescence bleed-through.

In mammalian, PLIN5 has been extensively characterized as a key mediator of lipid droplet-mitochondria interactions, particularly in oxidative tissues such as heart, liver, and skeletal muscle [30,31]. In contrast, the sea cucumber genome encodes only a single PLIN family member, PLIN2, raising the possibility that *A. japonicus* PLIN2 may functionally substitute for multiple PLIN paralogs. Consistently, our findings reveal striking functional parallels between *A. japonicus* PLIN2 and mammalian PLIN5, including regulation of starvation-induced lipid droplet-mitochondria contacts and promotion of fatty acid transfer to mitochondria. These observations suggest that the ability of PLIN proteins to mediate lipid droplet-mitochondria crosstalk is evolutionarily conserved, even though the specific PLIN family member fulfilling this role varies across species.

Accumulating studies reveal that lipid droplets exert a protective role in alleviating ER stress by sequestering and facilitating the clearance of misfolded proteins [32,33,34]. In accordance with these observations, our results show that OA-induced lipid droplet formation attenuates *V. splendidus*-induced ER stress, supporting a role of lipid droplets in maintaining ER homeostasis. Importantly, we further identify *A. japonicus* PLIN2 as a key mediator of lipid droplet-dependent ER stress alleviation. Knockdown of *A. japonicus* PLIN2 abolished the suppressive effect of lipid droplets on ER stress-related gene expression, indicating that lipid droplet accumulation alone is insufficient to maintain ER homeostasis in the absence of proper lipid droplet surface organization. This observation aligns with findings from mammalian systems, where PLIN2-dependent lipid storage suppressed cytotoxic ER stress responses in clear-cell renal cell carcinoma [35], whereas PLIN2 knockdown induces ER stress in human β-cells [36]. Mechanistically, as a major lipid droplet coat protein, *A. japonicus* PLIN2 may facilitate functional ER-lipid droplet contacts by stabilizing lipid droplet architecture, regulating lipid flux, or organizing protein assemblies at contact sites. Further studies are needed to elucidate how *A. japonicus* PLIN2 coordinates ER-lipid droplet interactions and modulates ER stress signaling pathways.

## 4. Materials and Methods

### 4.1. Cell Culture and Treatment

Coelomocytes were cultured as previously described [37]. Briefly, sea cucumbers were dissected under sterile conditions, and coelomic fluid was collected and immediately mixed with an equal volume of anticoagulant solution. The cell suspension was centrifuged at 800× *g* for 5 min at 16 °C, and the pellet was washed three times with isotonic buffer. Cells were then resuspended in Leibovitz’s L-15 medium supplemented with 100 μg mL^−1^ gentamicin and 100 U mL^−1^ penicillin-streptomycin. Cell density was determined using a hemocytometer with a 5 μL aliquot. The cells were subsequently adjusted to 1 × 10^6^ cells mL^−1^, seeded into culture plates, and incubated at 16 °C.

For *V. splendidus* infection, coelomocytes were transfected with siNC or si*plin2* for 24 h. The cells were then incubated with 500 μM oleic acid (OA; purity ≥ 99%) (Sigma Aldrich, St. Louis, MO, USA; O1008) conjugated to fatty acid free BSA (Equitech-Bio, Kerrville, TX, USA; BAH66) for 12 h. Subsequently, cells were infected with *V. splendidus* (MOI = 10) for an additional 12 h, after which they were collected for further analyses.

### 4.2. Phylogenetic Analysis

Protein sequences used for phylogenetic analysis were retrieved from the NCBI GenBank database (www.ncbi.nlm.nih.gov). Multiple sequence alignment was performed in MEGA5 using the ClustalW algorithm with default parameters. Phylogenetic trees were constructed using the maximum likelihood method with 1000 bootstrap replicates. The optimal substitution model was selected based on the lowest Bayesian Information Criterion value as implemented in MEGA. The robustness of the inferred tree topology was evaluated using bootstrap support values. The final tree was visualized, modified, and annotated using ChiPlot (v2.6.1) [38].

### 4.3. Quantification of Gene Expression

Total RNA extraction, cDNA synthesis, and quantitative real-time PCR (qRT-PCR) were conducted according to the manufactures’ instructions. Primer sequences are listed in Supplementary Table S1. β-actin, glyceraldehyde-3-phosphate dehydrogenase (GAPDH), and β-tubulin were evaluated as candidate reference genes for normalization of gene expression in coelomocytes under different treatments. The stability of these reference genes was assessed using the NormFinder and geNorm algorithms [39,40]. No significant variation in β-actin expression was observed among treatments, indicating that β-actin was suitable as the reference gene in this study. Relative gene expression was calculated using the 2^−ΔΔCT^ method, as previously described [41].

### 4.4. RNA Interference Assay

Specific small interfering RNAs (siRNAs) targeting *plin2* and thioredoxin reductase 2 (*trxr2*), along with a negative control siRNA (siNC), were designed and synthesized by GenePharma (Shanghai, China). The knockdown efficiency is shown in Supplemental Figure S1. RNA interference assay was performed as described in our previous study with minor modifications [42]. Briefly, coelomocytes seeded in 6-well plates were transfected with 1 μL of 20 μM siRNA using Lipo8000. Cells were then collected for further analysis.

### 4.5. Plasmid Construction

PCR amplification was performed using primers listed in Supplementary Table S1 to construct plasmids. Specifically, for fluorescence staining, the open reading frame of PLIN2 from *A. japonicus* were amplified and inserted into the pEGFP-N3 vector. An AH-deficient PLIN2 (ΔAH) constructure was generated using a mutagenesis kit (Vazyme, Nanjing, China; C214-01) following the manufacturer’s instructions and subsequently inserted into the same vector. For pull down assays, the coding sequences of endoplasmic reticulum resident protein 44 (ERP44) and TRXR2 were amplified and subcloned into the pET-32a vector to generate His-tagged constructs, while PLIN2 was additionally cloned into the pGEX vector to generate a GST-tagged construct. For co-immunoprecipitation assays, the coding sequences of ERP44 and TRXR2 were amplified and subcloned into the pcDNA3.1 vector. HA tags were introduced into both constructs using specific primers (Supplementary Table S1). Prior to transfection, all plasmids were purified using an Endo-free-Plasmid Mini Kit I (Omega, Norcross, GA, USA; D6948-01B) and verified by Sanger sequencing (Sangon Biotech, Shanghai, China).

### 4.6. Pull-Down and Protein–Protein Interaction Assays

Pull-down assays were performed to identify potential PLIN2-interacting proteins. Recombinant GST-PLIN2 was immobilized on GST-Sepharose resin (Sangon, Shanghai, China; C600031) by incubation on ice for 30 min, followed by three washes with wash buffer. The beads were then incubated with coelomocyte protein extracts overnight at 4 °C. GST alone, incubated with an equal amount of protein extracts under the same conditions, served as a control. After incubation, the resin was washed ten times with wash buffer to remove nonspecifically bound proteins, and bound proteins were eluted using elution buffer. Eluted proteins were separated by SDS-PAGE, and bands unique to the GST-PLIN2 sample were excised for LC-MS/MS analysis.

To validate the interactions in vitro, recombinant PLIN2-interacting proteins (ERP44 and TRXR2) were expressed in *E. coli* using the pET-32a vector. For GST pull-down assays, purified GST-PLIN2 was incubated with His-ERP44 or His-TRXR2 at a 1:1 molar ratio for 6 h at 4 °C, followed by incubation with GST-Sepharose resin overnight at 4 °C. The resin was then washed five times with wash buffer, eluted with elution buffer, and analyzed by SDS-PAGE. Reciprocal His pull-down assays were performed by incubating purified His-ERP44 or His-TRXR2 with GST-PLIN2 under the same conditions, followed by capture with His-affinity resin (Sangon, Shanghai, China; C600033), washing, elution, and SDS-PAGE analysis.

### 4.7. Fluorescence Staining and Confocal Imaging

For subcellular localization of PLIN2 and its AH-deficient PLIN2 variant (ΔAH), HEK293T cells (1.0 × 10^5^ cells per well) were seeded onto coverslips in 24-well plates. The following day, cells were transiently transfected with the PLIN2-EGFP or ΔAH -EGFP vector using Lipo8000 reagent (Beyotime, Shanghai, China; C0533). After 24 h, cells were fixed with 4% paraformaldehyde (PFA), followed by staining with LipidTOX Red (Thermo Fisher Scientific, Waltham, MA, USA; H34476) to label lipid droplets, followed by nuclear staining with DAPI.

To evaluate the role of PLIN2 in lipid droplet formation, coelomocytes (1.0 × 10^5^ cells per well) were seeded onto coverslips and transfected with siNC or si*plin2*. After 24 h, cells were incubated with 500 μM OA for an additional 12 h. Cells were then fixed with 4% PFA, stained with BODIPY 493/503 (Thermo Fisher Scientific, Waltham, MA, USA; D3922) to visualize lipid droplets, and counterstained with DAPI.

For pulse-chase assay, coelomocytes were transfected with siNC or si*trxr2* for 24 h, followed by treatment with 500 μM OA for 12 h. Cells were then incubated with BODIPY 558/568 C_12_ for 30 min (pulse) to label lipid droplet-associated fatty acids. After removal of excess probe by washing three times with PBS, cells were incubated in Leibovitz’s L-15 medium for 6 h to allow fatty acid mobilization. At the end of the chase, cells were fixed with 4% PFA and stained with Mito-Tracker Green (Beyotime, Shanghai, China; C1996S) to visualize mitochondria, and counterstained with DAPI.

In the above experiments, coverslips were mounted on glass slides using PBS containing 2.3% 1,4-diazabicyclo[2.2.2]octane and 50% glycerol. Fluorescence images were acquired using a TCS SP8 confocal laser scanning microscope (Leica, Wetzlar, Germany) at appropriate magnifications.

### 4.8. Flow Cytometry

HEK293T cells were transfected with the PLIN2-EGFP expression plasmid. After 24 h, cells were treated with OA or T863 for 12 h, harvested, and analyzed by flow cytometry using the FITC channel (NovoCyte, Santa Clara, CA, USA, Agilent) to quantify EGFP fluorescence intensity.

### 4.9. Cellular Triglyceride Assay

Coelomocytes cultured in 10 cm dishes were transfected with siNC or si*plin2*. After 24 h, cells were treated with OA for an additional 12 h. Cells were then collected, washed, and resuspended in PBS. The cell suspension was lysed by ice-cold sonication, and the resulting homogenate was used to measure triglyceride content according to the manufacturer’s instructions (Nanjing Jiancheng Bioengineering, Nanjing, China; A110-1-1).

### 4.10. ATP Assay

Coelomocytes were transfected with siNC or si*trxr2* for 24 h, followed by incubation with 500 μM OA. After washing with PBS, cells were incubated in Leibovitz’s L-15 medium for 6 h. Intracellular ATP content was measured using the Luminescent ATP Detection Assay Kit (Abcam, Cambridge, UK; ab113849) following the manufacturer’s instructions. Briefly, 50 μL of detergent was added to each well, and the 96-well plate was shaken to lyse the cells and stabilize ATP. Subsequently, 50 μL of substrate solution was added to each well, and the plate was shaken for 5 min, covered, and incubated in the dark for 10 min before luminescence measurement.

### 4.11. Co-Immunoprecipitation and Western Blot

HEK293T cells cultured in 10 cm dishes were co-transfected with PLIN2-EGFP and ERP44-HA or TRXR2-HA plasmids for 48 h. Cells were then harvested and lysed in immunoprecipitation lysis buffer (Beyotime, Shanghai, China; P0013J). After centrifugation, the supernatants were incubated with anti-HA magnetic beads (MCE, Shanghai, China; HY-K0201), anti-FLAG magnetic beads (MCE, HY-K0207), or control IgG beads (Sigma-Aldrich, St. Louis, MO, USA; A0919) at 4 °C overnight with gentle rotation. Beads were washed, and the bound proteins were eluted with loading buffer for subsequent immunoblot analysis.

Protein concentrations were determined using an enhanced BCA Protein Assay Kit (Beyotime, Shanghai, China; P0010S). Equal amounts of protein were separated by SDS-PAGE and transferred onto nitrocellulose membranes. Membranes were blocked with nonfat milk and incubated with primary antibodies, and then with horseradish peroxidase (HRP)-conjugated secondary antibodies. Protein bands were visualized using the ChemiDoc™ XRS+ imaging system (Bio-Rad, Hercules, CA, USA).

### 4.12. Protein Structure Prediction and Interaction Analysis

The three-dimensional structure of PLIN2, ERP44, and TRXR2 was predicted using AlphaFold3 (https://alphafoldserver.com/) [43]. Amino acid sequences obtained from *A. japonicus* cDNA were submitted to generate high-confidence structural models. Protein–protein interactions were modeled by docking PLIN2 with ERP44 or TRXR2 using the AlphaFold-Multimer module. Model confidence was assessed using the predicted local distance difference test (pLDDT) and predicted aligned error (PAE). Interface regions with pLDDT values greater than 70 and inter-domain PAE values lower than 5 Å were considered to indicate higher confidence in the predicted interaction interfaces. Complexes were visualized and analyzed in PyMOL2 (Schrödinger, LLC, New York, NY, USA) to examine potential interaction interfaces, hydrogen bonds, and surface complementarity, enabling identification of key residues involved in PLIN2-ERP44/TRXR2 interactions. Figures illustrating structural models and interaction sites were prepared using PyMOL2.

### 4.13. Prediction of Amphipathic Helices

Amphipathic helices (AHs) in PLIN2 were predicted using the Phyre2 protein structure prediction server (https://www.sbg.bio.ic.ac.uk/~phyre2/html/page.cgi?id=index). Each identified α-helix was further analyzed with the HeliQuest tool (https://heliquest.ipmc.cnrs.fr/cgi-bin/ComputParamsV2.py) to determine its helical wheel orientation. Helices exhibiting a clear segregation into a hydrophobic face of substantial length and a polar face enriched in positively charged residues were considered potential AHs. Selection criteria for AHs included hydrophobicity (H) between 0.3 and 0.6, hydrophobic moments (μH) > 0.4, and a slightly positive net charge.

### 4.14. Statistical Analysis

All experiments were independently performed at least three times. Data were analyzed using GraphPad Prism 8.0.1 and are presented as mean ± SD. Differences between two groups were analyzed using Student’s *t*-test, while differences among three or more groups were evaluated by one-way ANOVA followed by Tukey’s or Dunnett’s multiple comparisons test. A *p* value < 0.05 was considered statistically significant.

## 5. Conclusions

**Figure 7:**
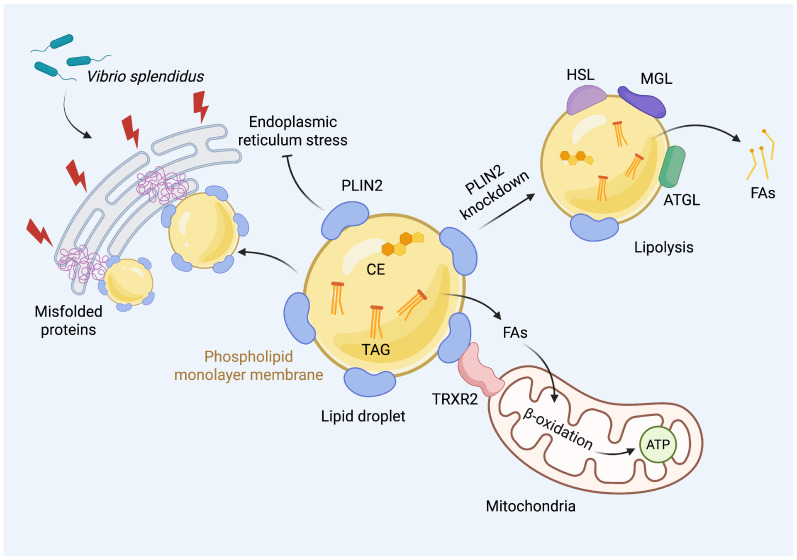
Roles of PLIN2 in coelomocytes of *Apostichopus japonicus*. PLIN2 localizes to lipid droplets and restricts access of lipases, such as ATGL, MGL, and HSL, to the droplet surface, thereby inhibiting excessive lipolysis. The interaction between PLIN2 and TRXR2 facilitates the transfer of fatty acid from lipid droplets to mitochondria for β-oxidation. Additionally, PLIN2 contributes to the alleviation of *Vibrio splendidus*-induced ER stress. ATGL, adipose triglyceride lipase; CE, cholesteryl ester; FAs, fatty acids; HSL, hormone-sensitive lipase; MGL, monoacylglycerol lipase; PLIN2, perilipin 2; TAG, triacylglycerol.

In conclusion, our study demonstrates that PLIN2 serves as a multifunctional lipid droplet-associated protein in *A. japonicus*, coordinating lipid storage, inter-organelle communication, and ER stress alleviation (Figure 7). By stabilizing lipid droplets, regulating lipid flux to mitochondria, and facilitating ER-lipid droplet interactions, PLIN2 preserves cellular energy homeostasis and mitigates stress responses. These findings highlight a conserved and expanded functional role of PLIN2 in marine invertebrates, providing new insights into the evolutionary adaptation of PLINs in regulating lipid droplet biology across eukaryotes.

## Data Availability

The raw data supporting the conclusions of this article will be made available by the authors on request.

## Supplementary Materials

The following supporting information can be downloaded at: https://www.mdpi.com/article/10.3390/ijms27114859/s1.

## Abbreviations

The following abbreviations are used in this manuscript:

atf6	cyclic AMP-dependent transcription factor atf-6 alpha
atgl	adipose triglyceride lipase
bip	endoplasmic reticulum chaperone bip
chop	DNA damage-inducible transcript 3 protein
cpt1α	carnitine O-palmitoyltransferase 1
ERP44	endoplasmic reticulum resident protein 44
hsl	hormone-sensitive lipase
ire1α	serine/threonine-protein kinase/endoribonuclease ire1α
mgl	monoacylglycerol lipase
OA	oleic acid
perk	eukaryotic translation initiation factor 2-alpha kinase 3
PLIN2	perilipin 2
PPARA	peroxisome proliferator-activated receptor alpha
TRXR2	thioredoxin reductase 2, mitochondrial
xbp1	x-box-binding protein 1
ΔAH	AH-deficient PLIN2
