# Supplementary material for: Perilipin 2 Stabilizes Lipid Droplets and Coordinates Mitochondrial Fatty Acid Flux and ER Stress Adaptation in Apostichopus japonicus
Source: Int J Mol Sci. 2026 May 28;27(11):4859. doi: 10.3390/ijms27114859 (PMC13256177; doi:10.3390/ijms27114859)
Supplement: Supplementary file 1 [file ijms-27-04859-s001.zip › ijms-4325990-supplementary.pdf]

Article

Perilipin 2 Stabilizes Lipid Droplets and Coordinates Mitochondrial Fatty Acid Flux and ER Stress Adaptation in *Apostichopus japonicus*

Hong Fan [1,†], Jintao Yu [2,†], Wenhao Wang [3], Zhimeng Lv [1], Si Zhu [1,*] and Chenghua Li [1,4,*]

1 State Key Laboratory of Agricultural Products Safety, Ningbo University, Ningbo 315211, China; 15669178030@163.com (H.F.); lvzhimeng@nbu.edu.cn (Z.L.)
2 School of Business, Ningbo University, Ningbo 315211, China; yjt6636@126.com
3 Yantai Marine Economic Research Institute, Yantai 264000, China; wangwenhao1128@163.com
4 Laboratory for Marine Fisheries Science and Food Production Processes, Qingdao National Laboratory for Marine Science and Technology, Qingdao 266237, China
* Correspondence: zhusi@nbu.edu.cn (S.Z.); lichenghua@nbu.edu.cn (C.L.); Tel.: +86-0574-87603877 (C.L.)
† These authors contributed equally to this work.

Supplemental Information

Supplemental Table S1, S2

Supplemental Figure S1, S2

Table S1 Primer pair sequences used in this study

Primer	Sequences (5'-3')	Application
atgl-F	CCACTTAGCCCTCGTTTT	qRT-PCR
atgl-R	TTCCGCCATCCACATACC	
hsl-F	CGAGAAGACAGCAGGGGTAA	
hsl-R	TCAAAGGCTCTAGGGAAAGG	
mgl-F	CCGAAGACACGGGCAAAAGT	
mgl-R	GAAGTGGGCCAGGAGAAACG	
pparα-F	GGAGAGGGGCAAAGTGGAAA	
pparα-R	CTTGGGCTAGAGGCCGTAAA	
cpt1α-F	TACCAGACAGGAAGGCGAAC	
cpt1α-R	ACCAGAGAAACGAGGCAAAT	
bip-F	GCTCACAAGCAATCCAGAAAACAC	
bip-R	CTTCTTGATGACCTTGAATGGGAG	
ire1α-F	CCACAGATGAGGCTGATTACGAGT	
ire1α -R	ATCTTGTGTAACCCTTCCCCTTGG	
perk-F	GAAATAAGACTCATTGGCAAAGGGG	
perk-R	TTAGCCAGGATGGTAACTTCTTGCG	
Atf6-F	GAATCCCCACCCCTGACTCCT	
Atf6-R	ATTTTGAGTCTGCGTGGTCGG	
xbp1-F	TGAAGAACAGAGTAGCTGCTCA	
xbp1-R	TGATGGATTCCATGTCCGCTGC	
chop-F	TTTGTATGTTGGCTTTATCACTGTCC	
chop-R	AGCCAGGATGGTAACTTCTTGCG	
β-actin-F	CCATTCAACCCTAAAGCCAACA	
β-actin-R	ACACACCGTCTCCTGAGTCCAT	

PLIN2-EGFP-F	GGTACCGCGGGCCCGGGATCCATGGAGGATATAAGCCACAACTCAG	
PLIN2-EGFP-R	CACCATGGTGGCGATGGATCCATTGCCATGCGGTAAATGAC	
ERP44-HA-F	AACTTAAGCTTGGTACCGAGCTCGATGAAGTCACTTTTGTTCATAACGTCT	
ERP44-HA-R	GAATTCCACCACACTGGACTAGTGTCAAGCGTAGTCTGGGACGTCGTATGGGTACAGCTCGTCACGTAGAATGGT	Co-IP
TRXR2-HA-F	AACTTAAGCTTGGTACCGAGCTCGATGTTGCAACAGATGGCTTCCG	
TRXR2-HA-R	GAATTCCACCACACTGGACTAGTGTCAAGCGTAGTCTGGGACGTCGTATGGGTA GCAACCGCTGACAGTAGG	
GST-PLIN2-F	GATCTGGTTCCGCGTGGATCCATGGAGGATATAAGCCACAACTCAG	
GST-PLIN2-R	CAGTCAGTCACGATGCGGCCGCCTAATTGCCATGCGGTAAATGAC	
His-ERP44-F	GCTGATATCGGATCCGAATTCATGAAGTCACTTTTGTTCATAACGTCT	
His-ERP44-R	TGGTGGTGGTGGTGCTCGAGTTACAGCTCGTCACGTAGAATGGT	Pull-down
His-TRXR2-F	GCTGATATCGGATCCGAATTCATGTTGCAACAGATGGCTTCCG	
His-TRXR2-R	TGGTGGTGGTGGTGCTCGAGTCAGCAACCGCTGACAGTAGG	
ΔAH-F	ACAGCTCGATATGCTGGAAGAAAAAGTACCCAT	Site-directed mutagenesis
ΔAH-R	TCCAGCATATCGAGCTGTGGCTGGGCAGTGTAAGCTG	
siPLIN2-F	GCAUCUCAAGUGUCUACCATT	
siPLIN2-R	UGGUAGACACUUGAGAUGCTT	RNA interference
siTRXR2-F	AGAGCUCCGUGUCUCAUAUTT	
siTRXR2-R	AUAUGAGACACGGAGCUCUTT	

atgl, adipose triglyceride lipase; *hsl*, hormone-sensitive lipase; *mgl*, monoacylglycerol lipase; *ppar*α, peroxisome proliferator-activated receptor alpha; *cpt1*α, carnitine O-palmitoyltransferase 1; *bip*, endoplasmic reticulum chaperone bip; *ire1*α, serine/threonine-protein kinase/endoribonuclease ire1α; *perk*, eukaryotic translation initiation factor 2-alpha kinase 3; *atf6*, cyclic AMP-dependent transcription factor atf-6 alpha; *xbp1*, x-box-binding protein 1; *chop*, DNA damage-inducible transcript 3 protein; PLIN2, perilipin 2; ERP44, endoplasmic reticulum resident protein 44; TRXR2, thioredoxin reductase 2, mitochondrial; ΔAH, AH-deficient PLIN2.

Table S2 Top five highest-scoring PLIN2-interacting proteins identified by mass spectrometry in coelomocytes

**Protein IDs**	**Protein**	**Score**	**Intensity**
A0A2G8K739	Putative endoplasmic reticulum resident protein 44 OS=*Stichopus japonicus* OX=307972 GN=BSL78_19324 PE=4 SV=1	323.31	679660000
A0A2G8JRZ2	Actin OS=*Stichopus japonicus* OX=307972 GN=BSL78_24675 PE=3 SV=1	127.1	139860000
A0A2G8LCM5	Thioredoxin reductase 2 OS=*Stichopus japonicus* OX=307972 GN=BSL78_05122 PE=3 SV=1	58.09	26343000
A0A2G8KRV7	Uncharacterized protein OS=*Stichopus japonicus* OX=307972 GN=BSL78_12380 PE=3 SV=1	48.933	35385000
A0AA51N2Q9	Gelsolin OS=*Stichopus japonicus* OX=307972 PE=2 SV=1	36.905	75162000

Figure S1

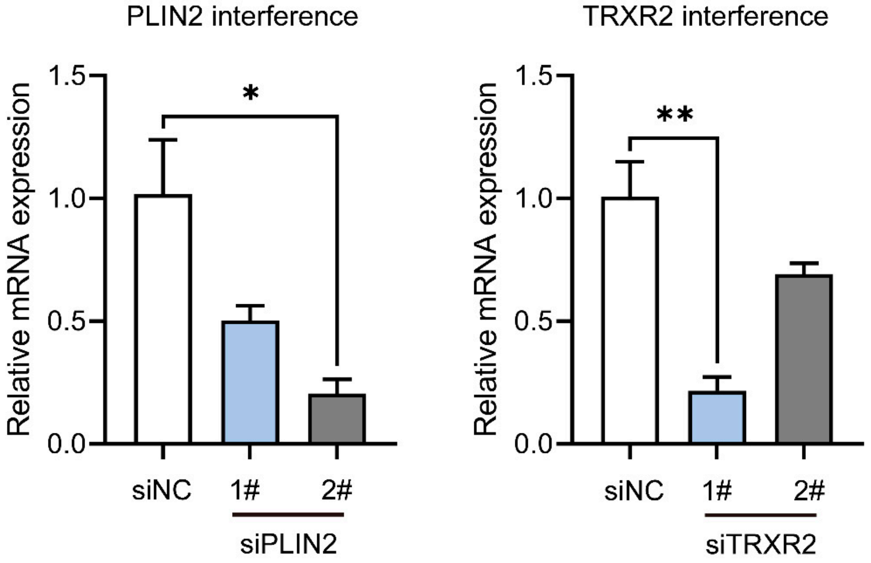

Figure S1 RNA interference. The knockdown efficiency of siPLIN2 and siTRXR2 was examined by qRT-PCR. Data from at least 3 independent experiments (mean ± SD). Significantly different experimental groups: *p<0.05, **p<0.01 by one-way ANOVA. siNC, negative control siRNA; PLIN2, perilipin 2; TRXR2, thioredoxin reductase 2, mitochondrial.

Figure S2

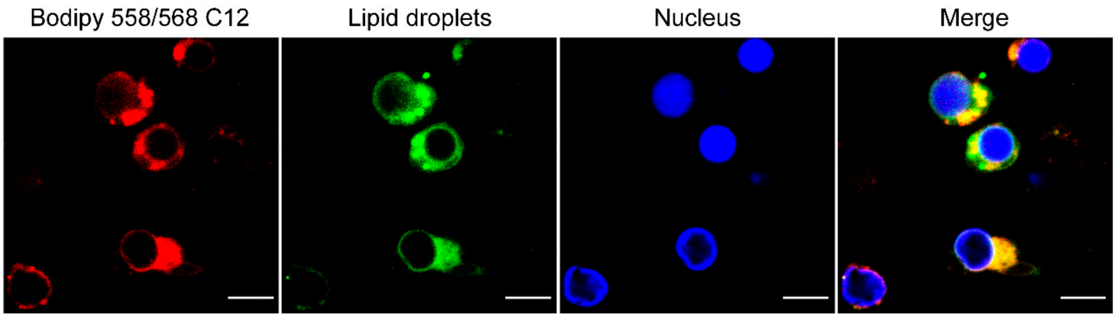

Figure S2 Colocalization of BODIPY 558/568 C_{12} and lipid droplets in coelomocytes at 0 h chase. Scale bar = 5 μM.
